# FeNi LDH/V_2_CT_x_/NF as Self-Supported Bifunctional Electrocatalyst for Highly Effective Overall Water Splitting

**DOI:** 10.3390/nano12152640

**Published:** 2022-07-31

**Authors:** Liming Yang, Tao Yang, Yafeng Chen, Yapeng Zheng, Enhui Wang, Zhentao Du, Kuo-Chih Chou, Xinmei Hou

**Affiliations:** 1 Beijing Advanced Innovation Center for Materials Genome Engineering, Collaborative Innovation Center of Steel Technology, University of Science and Technology Beijing, Beijing 100083, China; yangliming0619@163.com (L.Y.); yafeng_chen2013@163.com (Y.C.); zhengyapeng1994@163.com (Y.Z.); wangenhui@ustb.edu.cn (E.W.); kcc126@126.com (K.-C.C.); 2 MOE Key Laboratory of New Processing Technology for Non-Ferrous Metals and Materials, Guangxi Key Laboratory of Processing for Non-Ferrous Metals and Featured Materials, Guangxi University, Nanning 530004, China; zhentaodu@126.com

**Keywords:** V_2_CT_x_ MXene, layered double hydroxide, nanohybrids, overall water splitting

## Abstract

The development of bifunctional electrocatalysts with efficient oxygen evolution reaction (OER) and hydrogen evolution reaction (HER) is still a key challenge at the current stage. Herein, FeNi LDH/V_2_CT_x_/nickel foam (NF) self-supported bifunctional electrode was prepared via deposition of FeNi LDH on V_2_CT_x_/NF substrate by hydrothermal method. Strong interfacial interaction between V_2_CT_x_/NF and FeNi LDH effectively prevented the aggregation of FeNi LDH, thus exposing more catalytic active sites, which improved electrical conductivity of the nanohybrids and structural stability. The results indicated that the prepared FeNi LDH/V_2_CT_x_/NF required 222 mV and 151 mV overpotential for OER and HER in 1 M KOH to provide 10 mA cm^−2^, respectively. Besides, the FeNi LDH/V_2_CT_x_/NF electrocatalysts were applied to overall water splitting, which achieved a current density of 10 mA cm^−2^ at 1.74 V. This work provides ideas for improving the electrocatalytic performance of electrocatalysts through simple synthesis strategies, structural adjustment, use of conductive substrates and formation of hierarchical structures.

## 1. Introduction

Electrocatalytic water splitting to produce H_2_ and O_2_ as a green and efficient energy carrier has gained wide recognition and attention in the past decades [1,2,3,4]. Electrocatalytic water splitting consists of the process of HER and OER at the cathode and anode, respectively. In both cases, it is greatly significant to explore efficient and low-cost electrocatalysts to reduce the excessive overpotential [5,6]. To date, the commonly used OER and HER electrocatalysts are the commercially IrO_2_, RuO_2_ materials and Pt/C materials [7,8]; however, the high cost, limited reserves and poor multifunctional properties greatly limit their applications in water splitting [9,10].

At present, non-noble M-based materials like layered double hydroxide (LDH) have received extensive attention because of their flexible structural composition, diversified synthesis techniques and excellent electrocatalytic activity are considered to be outstanding electrocatalysts for water splitting [11,12,13,14,15,16,17]. Among LDH materials, FeNi LDH has received much attention for its good physicochemical properties, excellent OER electrocatalytic properties and durability in alkaline media [18,19]. Although FeNi LDH has been proved to be efficient in electrocatalysis, there are still two obstacles to further enhancements [20,21,22]. The first obstacle is its low conductivity due to poor carrier mobilities [23]. Two ways can be taken to overcome this obstacle. One is to combine FeNi LDH with highly conductive materials, such as graphene [24], crystal carbon conjugated (g-C_3_N_4_) [25], two-dimensional (2D) carbides and nitride (MXenes). The other is to grow FeNi LDH on a conductive skeleton, such as Al foil, Ni foam (NF), Ti sheet, carbon paper, carbon cloth and stainless steel mesh, etc [26,27]. The second obstacle is that aggregation of FeNi LDH leads to a reduction of its active site. There are some works that combined FeNi LDH and electric conducting graphene as a highly efficient electrocatalyst to enhance OER due to reducing aggregation [28,29]. Therefore, it is an effective method to introduce two-dimensional materials to enhance conductivity and increase restricted active sites of FeNi LDH.

As two-dimensional materials with a large specific surface area and high electrical conductivity [30], MXene has been extensively used in electrocatalyst applications in recent year [31,32,33,34]. In the MXene family, V_2_CT_x_ has various oxidation states of vanadium ion, so that the V surface layer may produce pseudocapacitive behavior [35], thus promoting the charge transfer between adsorbent and V_2_CT_x_ carrier. The previous work of our group has proved that the combination of V_2_CT_x_ and FeNi LDH can enhance the conductivity of FeNi LDH, reduce agglomeration phenomenon, and has excellent ORR and OER, which can be applied to a zinc–air battery [36]. To further explore and enhance the electrocatalytic performance of FeNi LDH, FeNi LDH/ V_2_CT_x_ was grown on NF to form a self-supporting electrode for efficient water splitting.

In this work, FeNi LDH/V_2_CT_x_/NF self-supporting electrode was obtained via one-step hydrothermal deposition of FeNi LDH nanosheets on V_2_CT_x_/NF substrate. Then, the properties of HER, OER and overall water splitting of FeNi LDH/V_2_CT_x_/NF were systematically studied. Lastly, the electrocatalytic mechanism of FeNi LDH/V_2_CT_x_/NF was explored by the density functional theory method.

## 2. Experimental Section

### 2.1. Materials

V_2_AlC (99 wt%, 400 mesh) was purchased from Foshan Xinxi Technology Co., Ltd, Guangdong, China. Nickel foam (NF) was purchased from Taiyuan Power Source Technology Co., Ltd, China. HF (~40 wt%), Fe(NO_3_)_3_·9H_2_O (96.8 wt%), Ni(NO_3_)_2_·6H_2_O (98.0 wt%), N-Methylpyrrolidone (NMP), tetrapropylammonium hydroxide (TPAOH, 40 wt%) and urea (CH_4_N_2_O, 96.0 wt%) were purchased from Aladdin, Shanghai, China. RuO_2_ (98.9 wt%), Pt/C (20 wt%) and Nafion solution (5 wt%) were purchased from Macklin, Shanghai, China.

### 2.2. Sample Preparation

#### 2.2.1. Preparation of V_2_CT_x_

Firstly, 1.0g V_2_AlC powder was gradually added to 20mL HF solution under stirring for 0.5 h and the suspension was kept stirring for 48 h at 55 °C. Secondly, the reaction mixture was rinsed with argon deoxygenated water and centrifuged. After that, the precipitate was re-dispersed to 20 mL TPAOH and stirred for 24 h. Next, the synthesized multilayer V_2_CT_x_ was centrifuged and washed twice, freeze-dried and weighed for V_2_CT_x_ MXene. V_2_CT_x_ nanosheets suspension concentration is 8 mg mL^−1^.

#### 2.2.2. Preparation of V_2_CT_x_/NF

NFs (1 × 3 × 0.1 cm^3^) were pretreated with concentrated HCl (3 M) for 0.5 h, and subsequently washed with acetone absolute ethanol and DI water. After that, NFs (1 × 3 × 0.1 cm^3^) were placed in 20 mL MXene suspension for 1 h. MXene is firmly adsorbed on the surface of NF by electrostatic adsorption, and V_2_CT_x_/NF is prepared. After repeated washing with Ar de-aerated DI water, vacuum drying for later use.

#### 2.2.3. Preparation of FeNi LDH/V_2_CT_x_/NF

FeNi LDH/V_2_CT_x_/NF was synthesized by the hydrothermal method. 100 mM urea, 5 mM Fe(NO_3_)_3_·9H_2_O and 15 mM Ni(NO_3_)_2_·6H_2_O were dissolved in 5 mL of DI water, stirring for 0.5 h to form a mixed solution (named solution A). 25 mL NMP was added to solution A and continued to stir for 0.5 h (named solution B). Then, V_2_CT_x_/NFs (1 × 3 × 0.1 cm^3^) were placed in solution B and heated for 12 h at 120 °C. For comparison, FeNi LDH/NF, V_2_CT_x_/NF and Ni(OH)_2_ V_2_CT_x_/NF was synthesized with the same process. The load of electrocatalyst was measured by weighing the NF electrode before and after electrocatalyst growth. In this report, for FeNi LDH/V_2_CT_x_/NF, the electrocatalyst load was about 5 mg cm^−2^.

#### 2.2.4. Preparation of RuO_2_/NF and Pt/C/NF

The RuO_2_ (10 mg) was dispersed ultrasonically in a mixture of isopropanol (750 μL), Nafion (16 μL, 5.0 wt%) and deionized water (250 μL). The suspension was ultrasonic for 30min to obtain an evenly dispersed ink solution. Then, 500 μL of electrocatalyst ink was loaded on 1 × 1 cm^2^ NF and the electrocatalyst load is about 5 mg cm^−2^. The process for preparing Pt/C/NF is the same, except that commercial Pt/C (20 wt%) is used instead of commercial RuO_2_.

### 2.3. Materials Characterization

The morphology was studied by field emission scanning electron microscopy (FESEM, FEI Zeiss Sigma 300). Transmission electron microscopy (TEM) was recorded using an FEI TalosF200x. Powder XRD pattern was obtained by Cu-Kα (λ = 0.1540 nm) on a SMARTLAB (9) X-ray photoelectron spectroscopy (XPS); the signal was captured using Axis Ultra DLD Kratos AXIS SUPRA.

### 2.4. Electrochemical Measurements

The electrocatalytic property was determined using a standard three-electrode system in 1.0 M KOH solution on a CHI 760E electrochemical workstation. FeNi LDH/V_2_CT_x_/NF served as the working electrode, carbon rod (OER) or platinum plate (HER) and Hg/HgO electrode (1 M KOH solution) were served as the counter electrode and reference electrode, respectively. According to the Nernst Equation (*E_RHE_* = *E_Hg/HgO_* + 0.059 pH + 0.098 V), all potentials measured at the Hg/HgO electrode in this study were converted to potentials with the reversible hydrogen electrode (RHE). OER and HER were measured under the condition of O_2_ and Ar saturation, respectively. Until the electrochemical data were tested, the working electrode was electrochemically activated for cyclic voltammetry 50 times. All the linear sweep voltammetry (LSV) curves are tested at 5 mV s^−1^ with 100% iR-compensation. The linear part of the Tafel curve was fitted according to the Tafel equation, and the Tafel slope was obtained to estimate the kinetic performance of the prepared electrocatalyst. Electrochemical impedance spectroscopy (EIS) was measured in the frequency range of 10^−2^ to 10^6^ Hz. The double layer capacitance (*C_dl_*) of the electrocatalysts was obtained by cyclic voltammetry at different scanning rates at non-Faraday potentials, and the ECSA of the catalysts was calculated. At room temperature, the prepared electrodes acted directly for overall water splitting in a two-electrode cell.

### 2.5. DFT Calculation Details

Vienna AB-Initio Simulation Package using the projector augmented wave (PAW) method was applied for all DFT calculations [37,38]. The local d-electrons of Ni and Fe in hydroxides were described by the DFT+U method. The value of U-J was 3.3 eV for V, 6.4 eV for Ni and 4.3 eV for Fe [9,39,40,41]. In order to simulate FeNi LDH/MXene nanohybrid, Fe doped NiOOH monolayer adsorption on O end V_2_CT_x_ was considered to simulate the electrocatalyst [42]. Since it is difficult to measure the crystal orientation of Fe-doped NiOOH intermediates, a surface exposed (001) Fe-doped NiOOH layer was coupled with V_2_CT_x_ to obtain the model with the most favorable energy structure and the lowest lattice mismatch for subsequent calculation. A 2 × 2 supercell of FeNi LDH and V_2_CT_x_ monolayer was constructed to become an 11.69 Å × 11.69 Å cell with a 16 Å vacuum space. Every four Ni atoms are replaced by one Fe atom. The whole calculation process uses the K-point grid with dimensions of 3 × 3 × 1. During the structure optimization, the tolerance of residual force for each atom is 0.05 eV/Å. When two consecutive self-consistent calculations are performed, the energy difference does not exceed 10^−4^ eV.

## 3. Results and Discussion

### 3.1. Preparation and Characterization of FeNi LDH/V_2_CT_x_/NF

**Figure 1:**
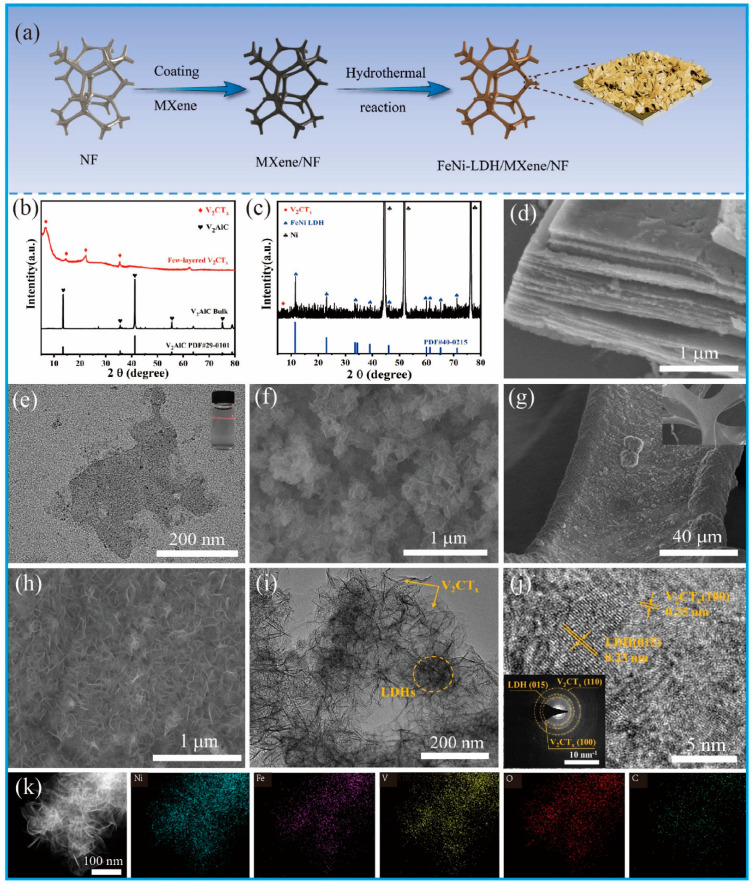
(**a**) Synthetic route of FeNi LDH/V_2_CT_x_/NF. SEM images of multilayered V_2_CT_x_ (**b**) XRD of V_2_AlC and V_2_CT_x_. (**c**) XRD patterns of FeNi LDH/V_2_CT_x_ peeled off the nickel foam. (**d**) with TEM images of few-layer V_2_CT_x_ (**e**), FeNi LDH/NF sample (**f**) and FeNi LDH/V_2_CT_x_/NF sample (**g**,**h**). TEM images of FeNi LDH/V_2_CT_x_ nanohybrids peeled from NF (**i**), HRTEM images of FeNi LDH/V_2_CT_x_ nanohybrids (**j**), the HAADF-STEM images in (**k**).

The synthesis scheme of FeNi LDH/V_2_CT_x_/NF is shown in Figure 1a. First, the Al layer of massive V_2_AlC powder was removed by 40 wt% HF selective etching to prepare multilayer V_2_CT_x_. After TPAOH intercalation and ultrasonic dissection, V_2_CT_x_ nanosheets with fewer layers and higher surface functional groups (−F, −OH) were obtained, which was beneficial to electrostatic adsorption on nickel foam and the nucleation and anchoring of FeNi LDH on V_2_CT_x_ surface. Then, NF was immersed in V_2_CT_x_ nanosheet suspension to obtain a V_2_CT_x_/NF electrode. After that, FeNi LDH nanosheets were hydrothermally deposited on V_2_CT_x_/NF substrate to obtain FeNi LDH/V_2_CT_x_/NF self-supported electrode. The XRD patterns of V_2_AlC and few-layer V_2_CT_x_ are illustrated in Figure 1b. The peaks of 41.3°and 13.5° correspond with the (103) and (002) crystal planes of V_2_AlC, respectively (PDF#29−0101). The peaks at 41.3°and 13.5° disappear completely for the exfoliated few-layer V_2_CT_x_, and the characteristic peak (002) is reduced to 6.74°, indicating that the Al atoms of few-layer V_2_CT_x_ are completely removed, and the C-lattice parameters increase [33,43,44]. In addition, the full and magnification XRD of the FeNi LDH/V_2_CT_x_/NF peeled off the nickel foam are shown in Figure S1 and Figure 1c. The peaks of 2*θ* = 44.5°, 51.9° and 76.37° correspond to the (111), (200), and (220) planes of Ni (PDF#04-0850). As shown in Figure 1c, the peaks at 2*θ* = 7.28° coincided very well with (002) crystal planes of V_2_CT_x_, while the peaks at 2*θ* = 11.41°, 22.974°, 34.425°, and 61.254° were in good agreement with the (003), (006), (012) and (113) of the FeNi LDH (PDF#40-0215), respectively; these results showed that FeNi LDH/V_2_CT_x_/NF had been successfully prepared.

The pristine V_2_AlC powders present bulk-like shapes (Figure S2) and unintercalated V_2_CT_x_ exhibits an accordion-like morphology (Figure 1d and Figure S3). V_2_CT_x_ nanosheets with a size of 200–300 nm and many edge defects were obtained by further intercalation and ultrasonic exfoliation of TPAOH (Figure 1e). As shown in Figure S4, the clear Tyndall effect in the supernatant illustrates the uniformly dispersed layers of V_2_CT_x_. Because of the electrostatic interaction between the positively charged NF substrate and the negatively charged V_2_CT_x_ surface, V_2_CT_x_ is able to anchor to the 3D NF structure, with silver NF turning into black NF (Figure S5). The low magnification SEM image of the FeNi LDH/V_2_CT_x_/NF sample can be seen in Figure 1g and the corresponding high magnification SEM image is shown in Figure 1h. The thickness of FeNi LDH nanosheets is about 8.03 nm (Figure S6). FeNi LDH is evenly distributed over the surface of the V_2_CT_x_/NF substrate. As a comparison, FeNi LDH was grown directly on NF without V_2_CT_x_ attached. It can be seen that FeNi LDH are clustered together (Figure 1f). The elemental mapping displays the well-distributed Fe, Ni, C and V elements in the FeNi LDH/V_2_CT_x_/NF (Figure S7); moreover, the TEM image of peeling FeNi LDH/V_2_CT_x_ from nickel foam (Figure 1i) indicates loose flake texture grows on the surface of V_2_CT_x_ with fewer layers and Figure 1j shows the interplanar spacing of 0.23 nm and 0.25 nm corresponding to (015) of FeNi LDH and the (100) of V_2_CT_x_, respectively. There are multiple diffraction rings on the (110) and (100) planes of V_2_CT_x_ and the (015) plane of FeNi LDH in the regional electron diffraction image selected in Figure 1j. The even distribution of V, Ni, Fe, O and C in the FeNi LDH/V_2_CTx nanohybrid is observed in Figure 1k.

**Figure 2:**
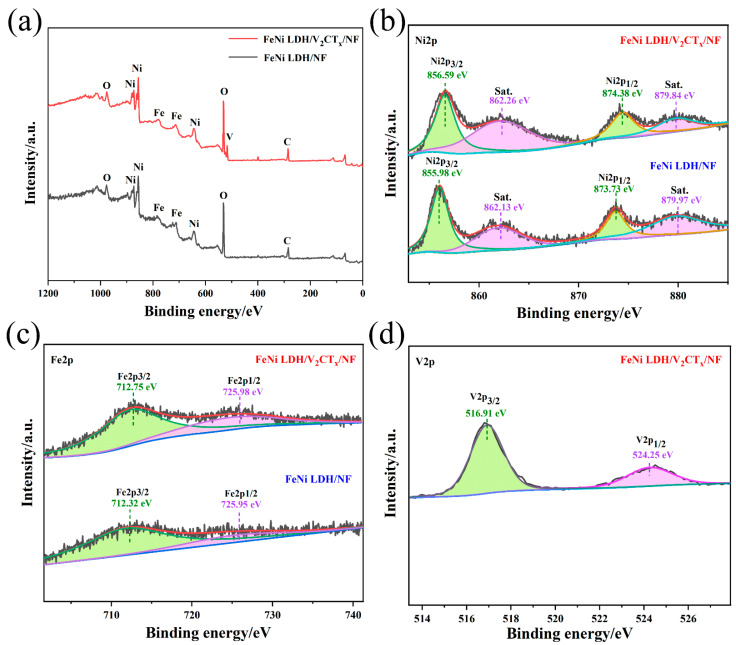
(**a**) Survey spectra of FeNi LDH/V_2_CT_x_/NF sample; (**b**,**c**) Ni 2p and Fe 2p XPS spectra of FeNi LDH/NF sample, respectively. (**d**) V 2p XPS spectra of FeNi LDH/V_2_CT_x_/NF.

The XPS spectra of the FeNi LDH/V_2_CT_x_/NF sample showed Fe, Ni, V, O and C signal peaks (Figure 2a). Since V_2_CT_x_/NF is covered by NiFe LDH, the signal peak of V is very weak. The high-resolution O 1s XPS spectra (Figure S8) were deconvolution at about 532.98 eV, 531.78 eV and 530.84 eV to obtain three component peaks corresponding to H-O-H, M-OH and M-O, respectively. In the Ni 2p XPS spectrum (Figure 2b), the two characteristic peaks of 2p_1/2_ and 2p_3/2_ orbits of Ni^2+^ are located at 874.38 eV and 856.59 eV, respectively [45]. Similarly, in Fe 2p spectra (Figure 2c), peaks at 725.98 eV and 712.75 eV are due to 2p_1/2_ and 2p_3/2_ orbits of Fe^3+^, respectively [46]. In contrast, for FeNi LDH/V_2_CT_x_/NF samples, the positive displacement of the Ni 2p peak is 0.6–0.65 eV and the Fe 2p peak is 0.43 eV. The higher the binding energy is, the higher the oxidation state of Fe and Ni iron after V_2_CT_x_ binding is. Specifically, the electron transfer from V_2_CT_x_ to LDHs increased the valence states of Fe and Ni, suggesting that there is a strong chemical interaction between FeNi LDH and V_2_CT_x_. Figure 2d shows the V 2p spectra, the V^4+^ of V_2_CT_x_ corresponds to the V 2p_1/2_ at 524.25 and V 2p_3/2_ at 516.91 eV [47].

### 3.2. Electrochemical Characterization

**Figure 3:**
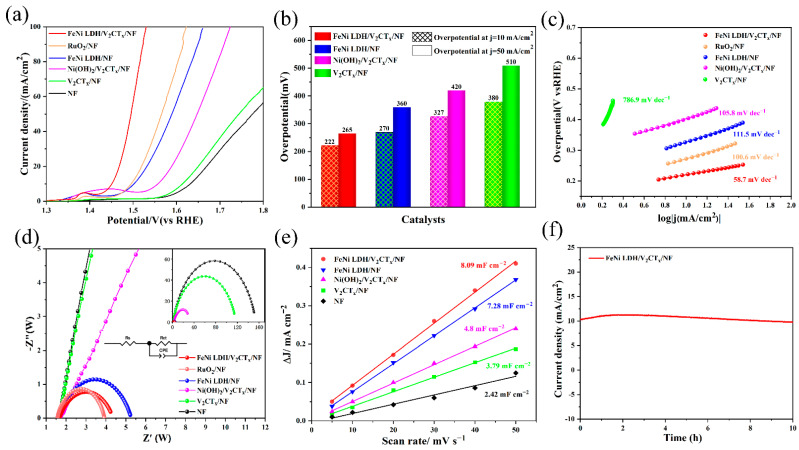
OER performances of prepared electrocatalysts. (**a**) LSV curves. (**b**) The overpotential of electrocatalysts. (**c**) Tafel plots. (**d**) EIS patterns. (**e**) *C_dl_* of samples. (**f**) Stability measurements of FeNi LDH/V_2_CT_x_/NF.

The OER electrocatalytic property of the prepared electrocatalyst was evaluated in 1.0 M KOH electrolyte with O_2_-saturated. The activation process of FeNi LDH/V_2_CT_x_/NF electrocatalyst is shown in Figure S9. After 50 cycles of voltammetry, the electrocatalyst is transformed from hydroxide to oxyhydroxides. The redox peaks of the Ni(II)/Ni(III) are located at the potential range of 1.25–1.55 V (vs. RHE) [48], which was the strongest at 1.39 V potential. As seen in Figure 3a, FeNi LDH/V_2_CT_x_/NF electrocatalyst with a minimum overpotential of 222 mV to achieve 10 mA cm^−2^ (η_10_ = 222 mV) exhibits highest catalytic activity, as compared with NF electrocatalysts (η_10_ = 400 mV), V_2_CT_x_/NF electrocatalysts (η_10_ = 380 mV), Ni(OH)_2_/V_2_CT_x_/NF electrocatalysts (η_10_ = 326 mV) and RuO_2_/NF (η_10_ = 265 mV); moreover, FeNi LDH/V_2_CT_x_/NF electrocatalyst achieves 50 mA cm^−2^ with the lowest overpotential of 265 mV (Figure 3b). Meanwhile, when comparing with the representative materials in Table S1 FeNi LDH/V_2_CT_x_/NF affords superior OER activity (η_10_ = 222 mV) to the excellent electrocatalysts reported recently, such as FeNi LDH/Ti_3_C2T_x_ (η_10_ = 298 mV) [15], CoNi-LDH/Ti_3_C_2_T_x_ (η_10_ = 257 mV) [49], H_2_PO^2−^/FeNi LDH/V_2_CT_x_ (η_10_ = 250 mV) [36] and N-CoFe LDH/NF (η_10_ = 233 mV) [50]. As seen in Figure 3c, the FeNi LDH/V_2_CT_x_/NF electrocatalyst shows the lowest Tafel slope of 58.7 mV dec^−1^, as compared with FeNi LDH/NF (111.5 mV dec^−1^), V_2_CT_x_/NF (786.9 mV dec^−1^), Ni(OH)_2_/V_2_CT_x_/NF (105.8 mV dec^−1^), NF (1708.6 mV dec^−1^) and commercial RuO_2_/NF (67.9 mV dec^−1^).

Figure 3d shows the elliptic semicircle of the EIS diagram, in which the sharp reduction of the semicircle diameter in the high-frequency region demonstrates the acceleration of charge transfer. The FeNi LDH/V_2_CT_x_/NF electrocatalyst has the lowest charge transfer impedance (*R_ct_* = 3.614 Ω) than that of Ni(OH)_2_/V_2_CT_x_/NF (*R_ct_* = 42.270 Ω) and FeNi LDH/NF (*R_ct_* = 4.275 Ω) electrocatalyst according to Table S2. The electrochemically active surface areas (ECSAs) were estimated from the *C_dl_* by cyclic voltammetry at 1.02–1.10 V (vs. RHE), as it is linear with ECSA (Figure S10). As shown in Figure 3e, the *C_dl_* value of FeNi LDH/V_2_CT_x_/NF is 8.09 mF cm^−2^, which is larger than those of FeNi LDH/NF (7.28 mF cm^−2^), Ni(OH)_2_/V_2_CT_x_/NF (4.8 mF cm^−2^) and V_2_CT_x_/NF (3.79 mF cm^−2^). Figure 3f shows the stability measurements of FeNi LDH/V_2_CT_x_/NF for η = 10 mA cm^−2^ at 0.222 V. The current density of FeNi LDH/V_2_CT_x_/NF did not decrease significantly after 10 h. After a 10h OER test, the morphology of FeNi LDH/V_2_CT_x_/NF has not changed significantly (Figure S11), which further confirms its high stability. In addition, XPS measurements of FeNi LDH/V_2_CT_x_/NF were performed (Figure S12). From the high-resolution Ni 2p spectra and Fe 2p spectra (Figure S11a,b), it can be seen that the characteristic peaks of both Ni 2p and Fe 2p have a slight rise. The former indicates that Ni^2+^ is oxidized to Ni^3+^, and the latter indicates that Fe is also partially oxidized, which is beneficial to accelerate the redox activity in the OER process; moreover, the two peaks of the V 2p spectrum (Figure S11c) are still located at 516.91 eV (V 2p_3/2_) and 524.25 eV (V 2p_1/2_), corresponding to the V^4+^ state, indicating its good stability during the OER.

**Figure 4:**
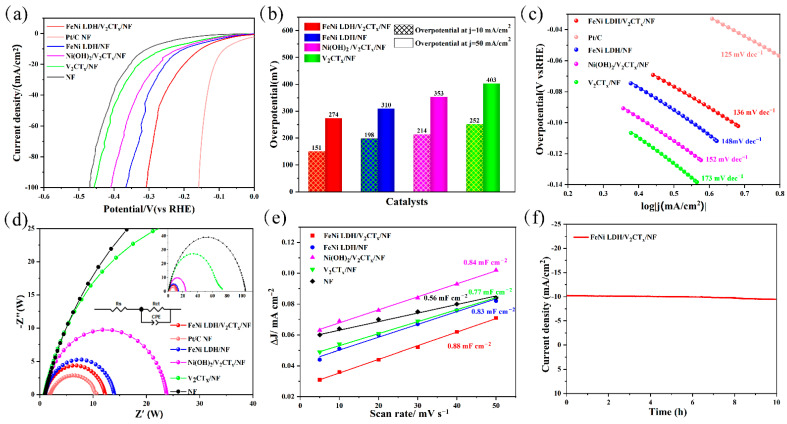
HER performances of prepared electrocatalysts. (**a**) LSV curves. (**b**) The overpotential of electrocatalysts. (**c**) Tafel plots. (**d**) EIS patterns. (**e**) *C_dl_* of samples. (**f**) Stability measurements of FeNi LDH/V_2_CT_x_/NF.

To further explore the HER activity, FeNi LDH/V_2_CT_x_/NF with 1 × 1 cm^2^ was served as the working electrode, while Pt plate, Hg/HgO (1 M KOH) and 1 M KOH were used as the counter electrode, reference electrode and electrolyte, respectively. The overpotential of FeNi LDH/V_2_CT_x_/NF at a current density of 10 mA cm^−2^ is 151 mV (η_10_ = 151 mV), which is smaller than that of FeNi LDH/NF (η_10_ = 198 mV), Ni(OH)_2_ LDH/V_2_CT_x_/NF (η_10_ = 214 mV), V_2_CT_x_/NF (η_10_ = 252 mV) and NF (η_10_ = 305 mV), except for Pt/C/NF (η_10_ = 81 mV) (Figure 4a). As seen from Figure 4b, FeNi LDH/V_2_CT_x_/NF electrocatalyst achieves a current density of 50 mA cm^−2^ with the lowest overpotential of 274 mV; moreover, the Tafel slope of the FeNi LDH/V_2_CT_x_/NF was 136 mV/dec, which was lower than that of FeNi LDH/NF (148 mV dec^−1^), Ni(OH)_2_ LDH/V_2_CT_x_/NF (152 mV dec^−1^) and V_2_CT_x_/NF (173 mV dec^−1^) (Figure 4c), further confirmed the internal reaction kinetics of the prepared FeNi LDH/V_2_CT_x_/NF significantly accelerated.

EIS was tested at −1.0 V (vs. RHE). The results (Figure 4d and Table S3) showed that FeNi LDH/V_2_CT_x_/NF had the lowest *R_ct_* (*R_ct_* = 10.85 Ω) of all electrocatalysts, indicating good reaction kinetics and faster charge transfer. The CV curves were obtained between 0.123 to 0.223 V (vs RHE) to estimate the ECSA of all electrocatalysts (Figure S13). FeNi LDH/V_2_CT_x_/NF measured much higher *C_dl_* (0.88 mF cm^−2^) than FeNi LDH/NF (0.83 mF cm^−2^), Ni(OH)_2_ LDH/V_2_CT_x_/NF (0.84 mF cm^−2^), V_2_CT_x_/NF (0.77 mF cm^−2^) and NF (0.56 mF cm^−2^) (Figure 4e). As shown in Figure 4f, the FeNi LDH/V_2_CT_x_/NF electrocatalyst maintained excellent catalytic performance and showed better durability in alkaline solution after a 10 h cycling test at −10 mA cm^−2^. As seen in Figure S14, the morphology of the electrocatalyst was well maintained after HER testing, indicating its good mechanical stability. Besides, Figure S15 shows the XPS spectra of Ni 2p, Fe 2p and V 2p in the FeNi LDH/V_2_CT_x_/NF before and after the durability test. No significant changes were observed, indicating high electrochemical stability.

**Figure 5:**
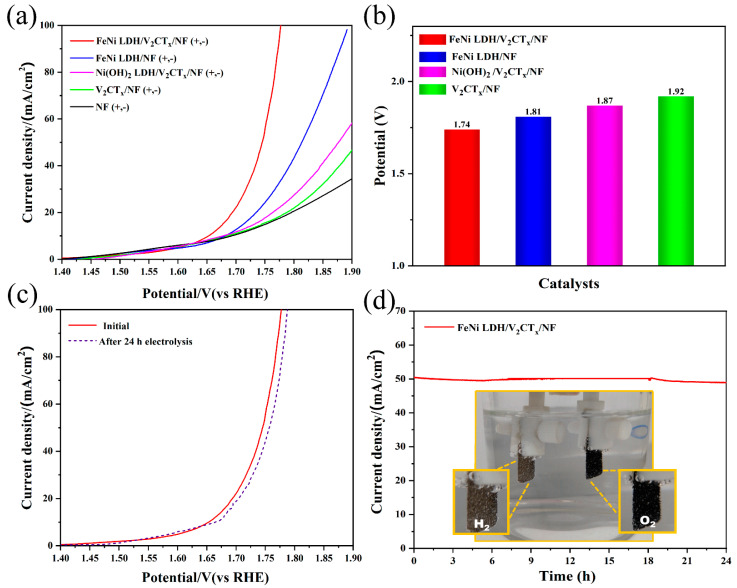
Overall water splitting performances of synthesized electrocatalysts. (**a**) LSV curves. (**b**) The voltage of electrocatalysts for overall water splitting. (**c**) LSV curves of FeNi LDH/V_2_CT_x_/NF before and after 15 h of electrolysis. (**d**) Stability measurements of FeNi LDH/V_2_CT_x_/NF. The inserted image shows the simultaneous production of H_2_ and O_2_ bubbles.

Based on the results, the FeNi LDH/V_2_CT_x_/NF electrode can be used as a robust and efficient electrocatalyst for OER and HER. Therefore, the FeNi LDH/V_2_CT_x_/NF electrode was used as both cathode and anode in 1.0 M KOH for overall water splitting. It can be seen from Figure 5a that when the current density is 50 mA cm^−2^, it is easy to achieve a voltage of 1.74 V (η_50_ = 1.74V), which is small than FeNi LDH/NF (η_50_ = 1.81 V), Ni(OH)_2_ LDH/V_2_CT_x_/NF (η_50_ = 1.87 V) and V_2_CT_x_/NF (η_50_ = 1.92 V) (Figure 5b); moreover, FeNi LDH/V_2_CT_x_/NF has a superiority compared with other electrocatalysts prepared by the one-step hydrothermal method in the literature (Table S4), such as NiFe LDH/NiCoP/NF (1.75 V) [14], NiCo_2_S_4_@NiFe-LDH/NF (1.83 V) [51] and NiCo-LDH/NF (1.86 V) [52]. As can be seen from the LSV curve before and after 24 h of the overall water splitting test (Figure 5c), the performance of the electrocatalyst is relatively stable and the overpotential changes little at 10 mA cm^−2^. Besides, the overall water splitting can be continued for 24 h steadily at 50 mA cm^−2^ (Figure 5d) and the O_2_ and H_2_ bubbles can be clearly observed (the inset of Figure 5d).

### 3.3. Mechanism Analysis

**Figure 6:**
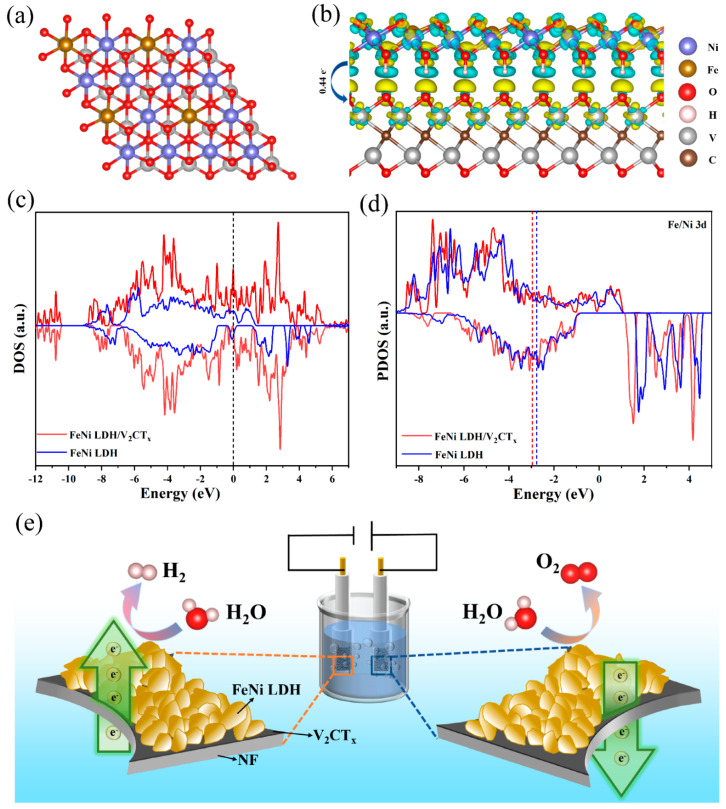
(**a**) Top view of FeNi LDH/V_2_CT_x_ composite. (**b**) Differential charge density in FeNi LDH/V_2_CT_x_ composite. (**c**) DOS of FeNi LDH/V_2_CT_x_ and FeNi LDH. The Fermi level is shifted to zero. (**d**) PDOS of FeNi LDH/V_2_CT_x_ and FeNi LDH. (**e**) Illustration of the FeNi LDH/V_2_CT_x_/NF for OER and HER.

To further understand the interaction of FeNi LDH and V_2_CT_x_, the first-principles calculation was performed based on the DFT method (Figure 6). The side and top view of model structure of FeNi LDH/V_2_CT_x_ composite after structural relaxation are shown in Figure 6a and Figure S16. Figure 6b shows differential charge density in FeNi LDH/V_2_CT_x_ composite study the charge redistribution by subtracting the charge densities of FeNi LDH and V_2_CT_x_ slabs from the total charge density of the structure, which the light blue and yellow colors mean electron loss and electron gain, respectively. After V_2_CT_x_ is introduced, because V_2_CT_x_ has stronger electronegativity than FeNi LDH, electrons are transferred from FeNi LDH to V_2_CT_x_. The bader quantitative analysis shows that the amount of electron transfer from FeNi LDH to V_2_CT_x_ is 0.44 e^-^ per unit cell, which may be due to the high electronegativity of V_2_CT_x_, which is easy to attract electrons from transition metal.

The project density of states (PDOS) and density of states (DOS) of FeNi LDH and FeNi LDH/V_2_CT_x_ are shown in Figure 6c,d. As exhibited in Figure 6c, compared with FeNi LDH, the electron density of FeNi LDH/V_2_CT_x_ near the Fermi level performs increased DOS and charge transfer, which is conducive to the adsorption–desorption performance of the reaction intermediates. Therefore, the conductivity of the electrocatalyst is improved and the energy barrier of OER is reduced [53,54]. As can be seen from Figure 6d, the PDOS of FeNi LDH/V_2_CT_x_ is further away from the Fermi energy level than that of FeNi LDH, where the D-band centers of FeNi LDH/V_2_CT_x_ and FeNi LDH are −2.96 eV and −2.77 eV, respectively. DFT simulations combined with experiments show that the increased activity is due to a modest downshift in the *E_d_* level, which regulates the desorption and adsorption capacities of the intermediates according to D-band center theory [55,56]. Therefore, the balance between intrinsic activity adsorption capacity of electrocatalyst to intermediate should be considered. As illustrated in Figure 6e, FeNi LDH/V_2_CT_x_ shows an excellent electrocatalytic performance because of the increase of active sites and electrical conductivity.

## 4. Conclusions

In summary, through a simple one-step hydrothermal method, a self-supported bifunctional FeNi LDH/V_2_CT_x_/NF electrode has been successfully prepared. The presence of V_2_CT_x_ promoted the dispersed growth of FeNi LDH nanosheets onto V_2_CT_x_/NF working electrode and reduced the obstacle of electron transfer. The NF scaffold provides a highly exposed active surface and facilitates the electrolyte infiltration and gas dissipation during OER. As a result, the overpotential of 222 mV and 151 mV of FeNi LDH/V_2_CT_x_/NF electrode at 10 mA cm^−2^ for the OER and the HER, respectively, proved an excellent electrocatalytic property. Besides, FeNi LDH/V_2_CT_x_/NF has a superiority (1.74 V) for overall water splitting at 50 mA cm^−2^ compared with other electrocatalysts prepared by one-step hydrothermal method in the literature; moreover, the current density of OER, HER and overall water splitting could remain almost the same after 10, 10 and 24 h test, underscoring the fundamentally improved of our bifunctional electrocatalysts.

## Data Availability

Not applicable.

## Supplementary Materials

The following supporting information can be downloaded at: https://www.mdpi.com/article/10.3390/nano12152640/s1 [57,58,59,60,61,62,63,64,65,66,67,68,69,70,71,72,73,74,75,76].
