# Supplementary material for: FeNi LDH/V2CTx/NF as Self-Supported Bifunctional Electrocatalyst for Highly Effective Overall Water Splitting
Source: Nanomaterials (Basel). 2022 Jul 31;12(15):2640. doi: 10.3390/nano12152640 (PMC9370147; doi:10.3390/nano12152640)
Supplement: Supplementary file 1 [file nanomaterials-12-02640-s001.zip › nanomaterials-1844220-supplementary.pdf]

Supplementary Information

FeNi LDH/V_2CT_x/NF as Self-Supported Bifunctional Electrocatalyst for Highly Effective Overall Water Splitting

Liming Yang [1], Tao Yang [1,*], Yafeng Chen [1], Yapeng Zheng [1], Enhui Wang [1], Zhentao Du [2], Kuo-Chih Chou [1] and Xinmei Hou [1,*]

[1] Beijing Advanced Innovation Center for Materials Genome Engineering, Collaborative Innovation Center of Steel Technology, University of Science and Technology Beijing, Beijing 100083, China; yangliming0619@163.com (L.Y.); yafeng_chen2013@163.com (Y.C.); zhengyapeng1994@163.com (Y.Z.); wangenhui@ustb.edu.cn (E.W.); kcc126@126.com (K.-C.C.)

[2] MOE Key Laboratory of New Processing Technology for Non-ferrous Metals and Materials, Guangxi Key Laboratory of Processing for Non-Ferrous Metals and Featured Materials, Guangxi University, Nanning 530004, China; zhentaodu@126.com

* Correspondence: yangtaoustb@ustb.edu.cn (T.Y.); houxinmeiustb@ustb.edu.cn (X.H.)

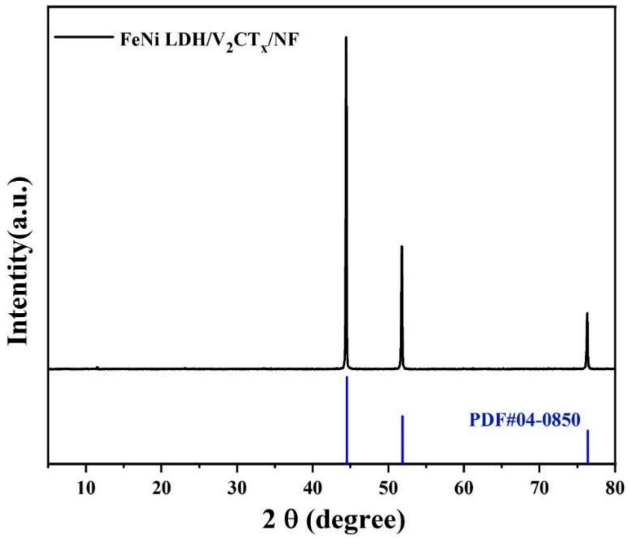

Figure S1. XRD patterns of FeNi LDH/V_2CT_x/NF.

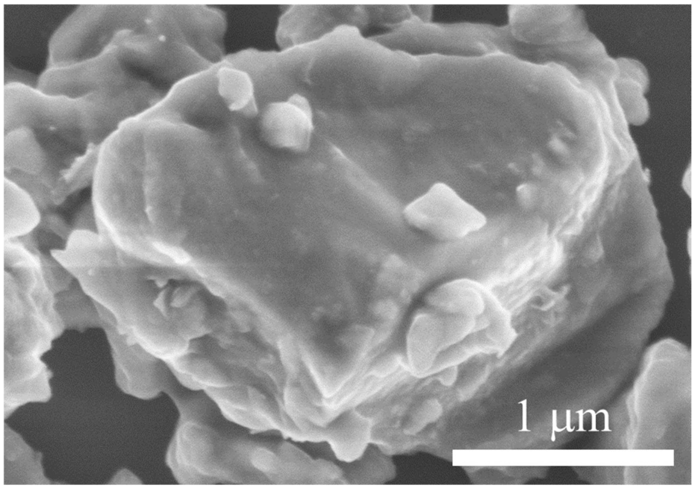

Figure S2. The SEM images of pristine V_2AlC powders.

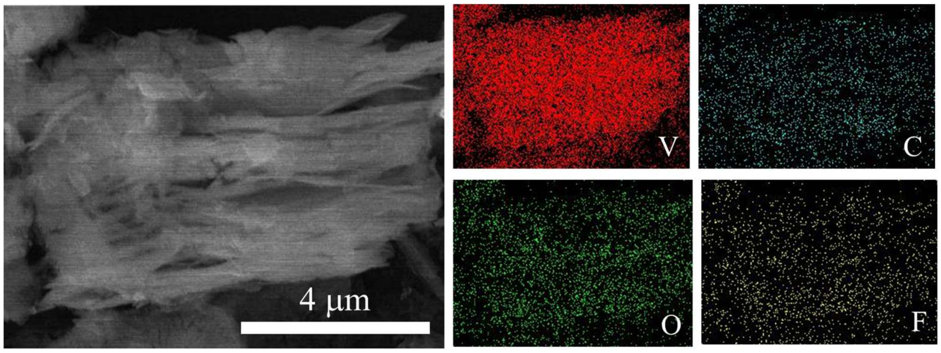

Figure S3. Elemental mapping showing the uniform distribution of V, C, O and F elements.

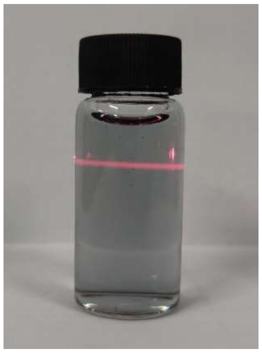

Figure S4. Optical image of V_2CT_x nanohybrids with the corresponding Tyndall effect.

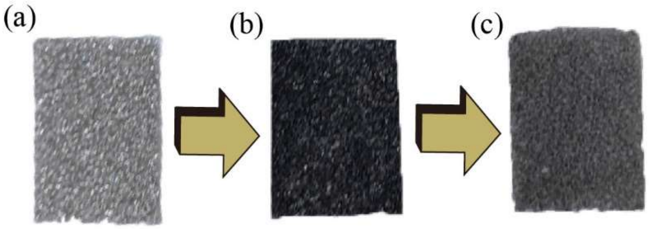

Figure S5. Optical image of (a) the bare NF, (b) the V_2CT_x/NF, (c) the FeNi LDH/V_2CT_x/NF in V_2CT_x nanohybrids.

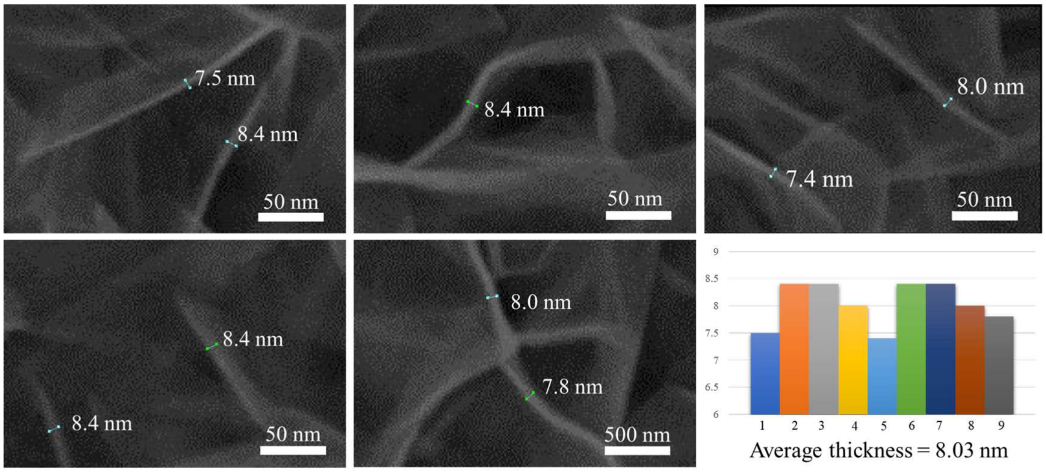

Figure S6. Average thickness of FeNi LDH nanosheets at different positions.

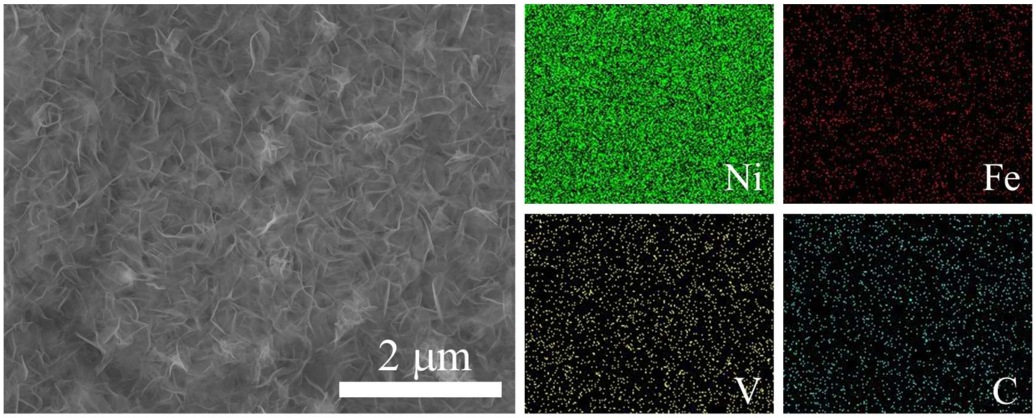

Figure S7. Elemental mapping showing the uniform distribution of Ni, Fe, V and C elements in FeNi LDH/V_2CT_x/NF nanohybrids.

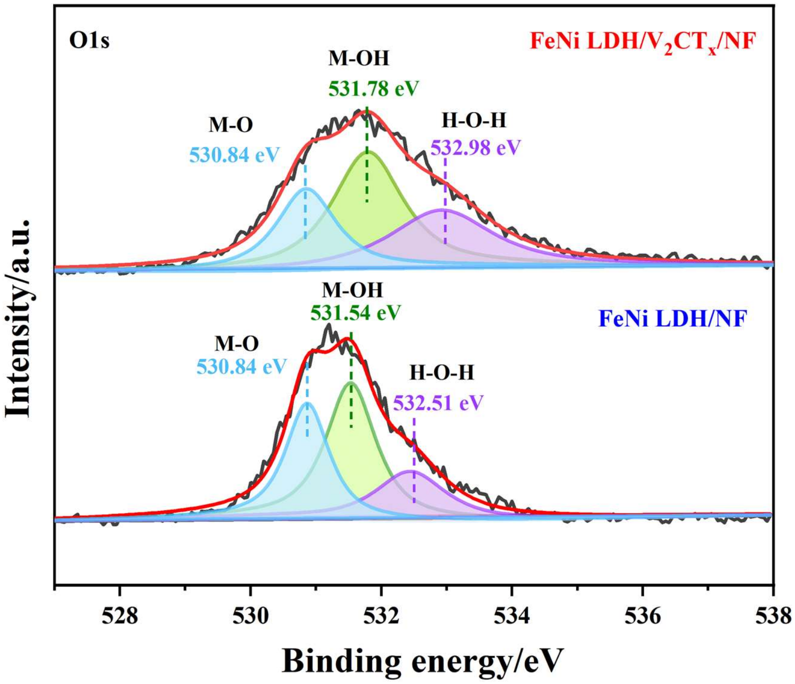

Figure S8. O 2p in obtained FeNi LDH/V_2CT_x/NF and FeNi LDH/NF.

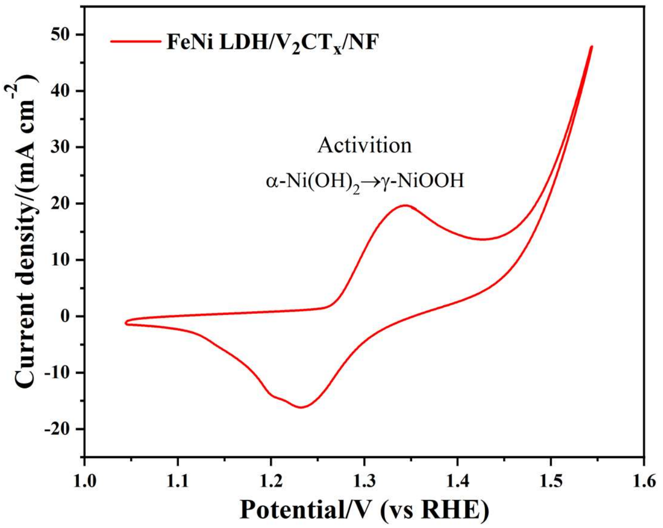

Figure S9. CV curves of FeNi LDH/V_2CT_x/NF at a scan rate of 50 mV s^{-1}.

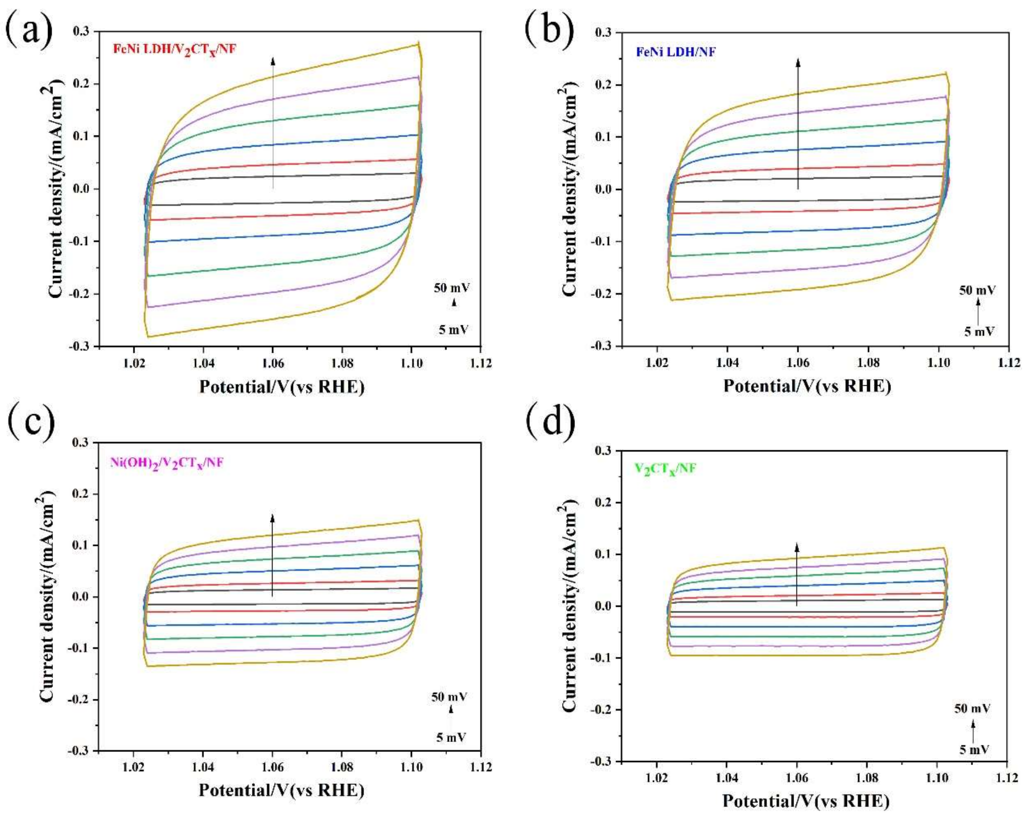

Figure S10. CV curves (1.02-1.10 V vs RHE) of (a) FeNi LDH/V_2CT_x/NF, (b) FeNi LDH/NF, (c) $Ni(OH)_2$ LDH/V_2CT_x/NF and (d) V_2CT_x/NF at various scan rate for OER.

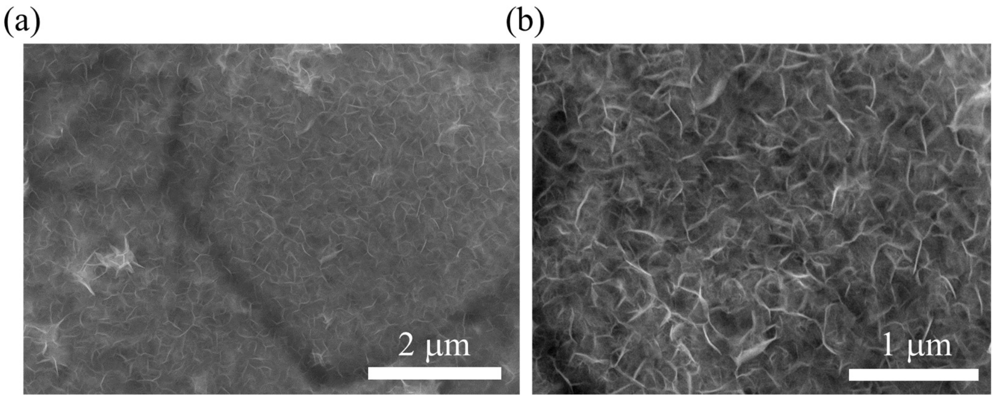

Figure S11. the SEM of FeNi LDH/V_2CT_x/NF after a 10h OER test.

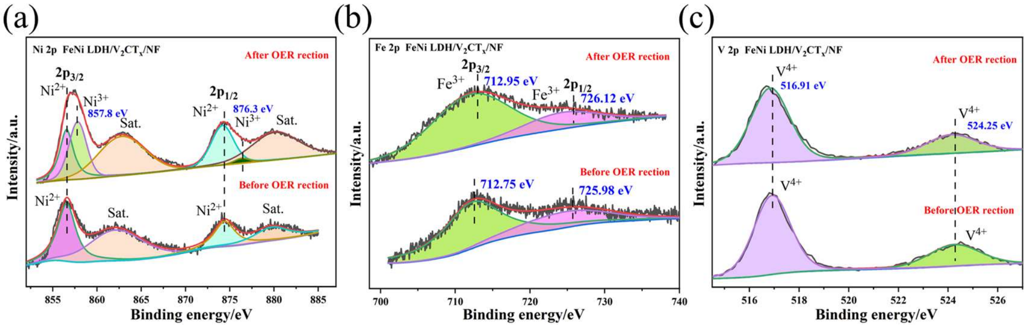

Figure S12. High-resolution XPS spectra of (a) Ni 2p, (b) Fe 2p and (c) V 2p in obtained FeNi LDH/V_2CT_x/NF and corresponding spectra after OER reaction.

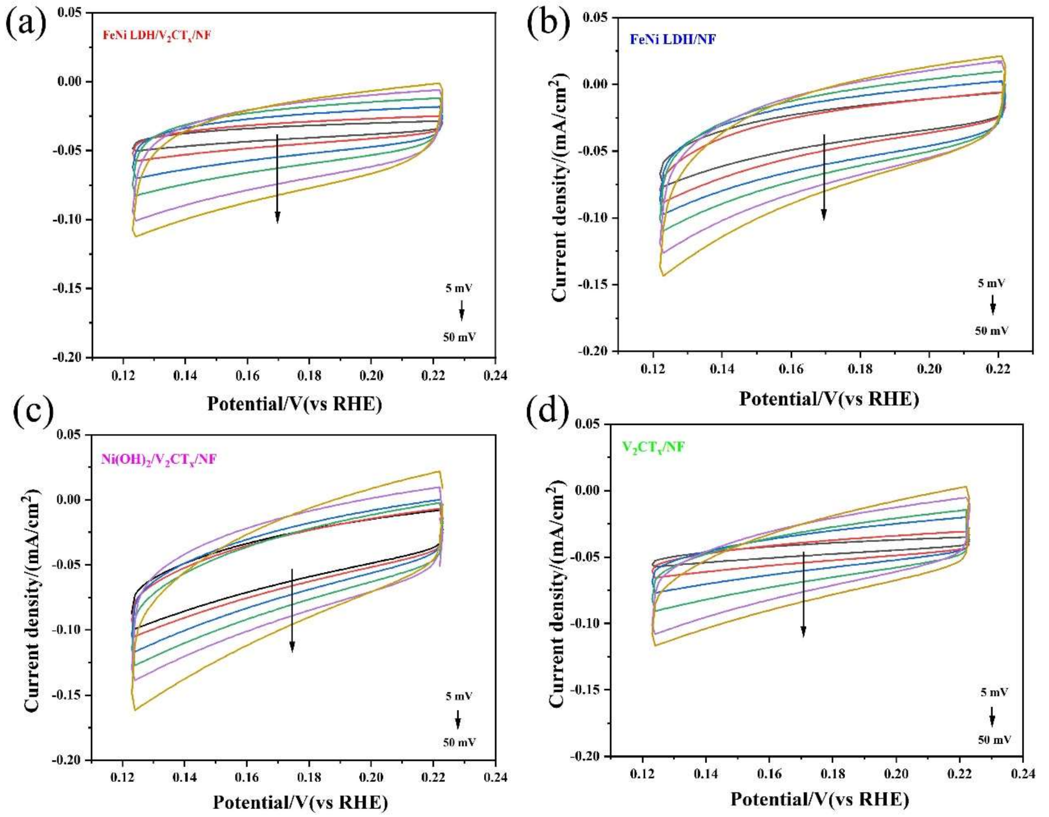

Figure S13. CV curves (0.123-0.223 V vs RHE) of (a) FeNi LDH/V_2CT_x/NF, (b) FeNi LDH/NF, (c) $Ni(OH)_2$ LDH/V_2CT_x/NF and (d) V_2CT_x/NF at various scan rate for HER.

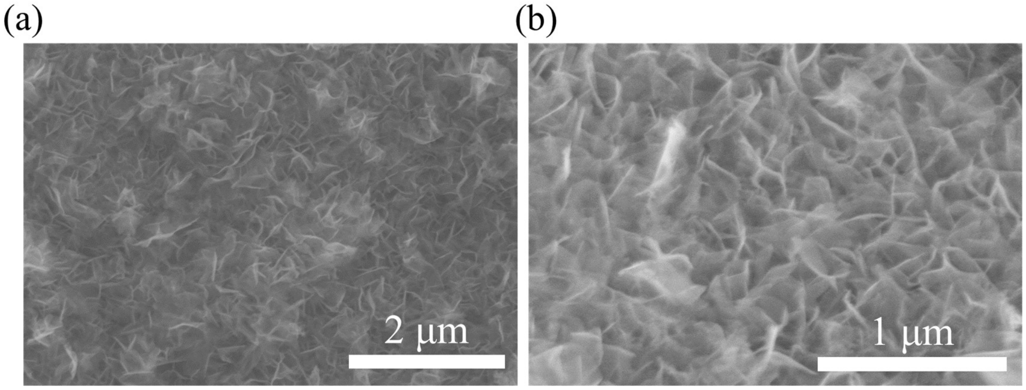

Figure S14. the SEM of FeNi LDH/V_2CT_x/NF after a 10h HER test.

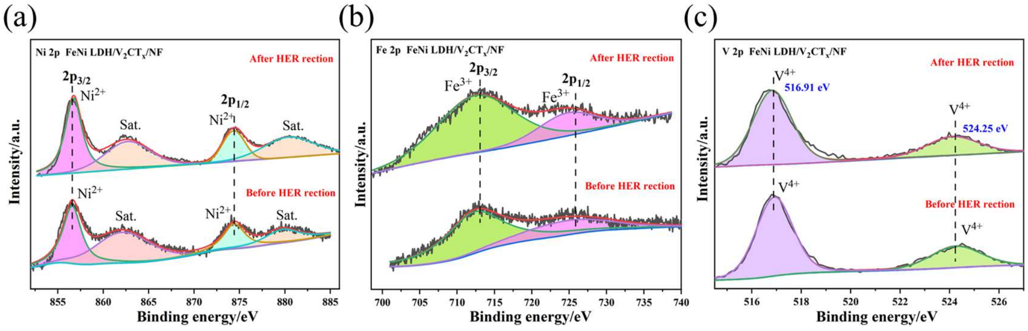

Figure S15. High-resolution XPS spectra of (a) Ni 2p, (b) Fe 2p and (c) V 2p in obtained FeNi LDH/V_2CT_x/NF and corresponding spectra after HER reaction.

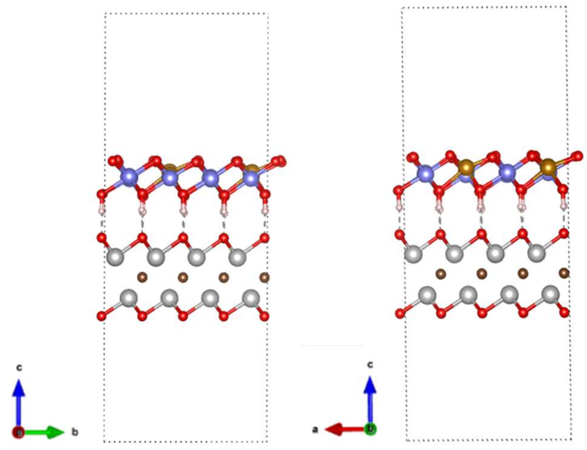

Figure S16. The side view of model structure of FeNi LDH/V_2CT_x composite.

Table S1. Comparison of η_{10} and Tafel slope OER between FeNi LDH/V_2CT_x/NF in this work and various Ni/Fe-based catalysts recently reported.

Materials	η=10 mV	Tafel slope	Ref.
**FeNi LDH/V_2CT_x/NF**	222	58.7	This work
**FeNi LDH/$Ti_3C_2T_x$**	298	43	[1]
**NiMn LDH/rGO**	280	46	[2]
**nNiFe LDH/3D MPC**	340	71	[3]
**NiMo/FG**	338	67	[4]
**N-CoFe LDH/NF**	233	40	[5]
**FeMnO NSs**	265	64	[6]
**$CoFe_2O_4$ NSs**	275	42	[7]
**CoNi-LDH/$Ti_3C_2T_x$**	257	68	[8]
**MOOH/$V_4C_3T_x$**	275	51	[9]
**NiFeCe LDH/$Ti_3C_2T_x$**	260	43	[10]
**Co_3O_4/$Ti_3C_2T_x$**	300	118	[11]
**FeCo LDH/$Ti_3C_2T_x$**	268	85	[12]
**MoO_4^{2-}/$NiCo_{1.5}Fe_{0.5}$ LDH**	239	43	[13]
**NiFeP/$Ti_3C_2T_x$**	286	35	[14]

**H_2PO^{2-}/FeNi LDH/V_2CT_x**	250	46.5	[15]
**MoS_2/NiFeCr LDH**	270	85	[16]
**CoFe LDH/$Ti_3C_2T_x$**	319	50	[17]
**NiCo LDH/GF**	249	108	[18]

Table S2. EIS parameters of synthesized catalysts for OER.

Materials	Rs(Ω)	CPE-T	CPE-P	Rct(Ω)
**FeNi LDH/V_2CT_x/NF**	1.656	0.061	0.719	3.614
**RuO_2/NF**	1.563	0.023	0.802	2.363
**FeNi LDH/NF**	1.407	0.098	0.744	4.275
**$Ni(OH)_2$/V_2CT_x/NF**	1.815	0.094	0.645	42.270
**V_2CT_x/NF**	0.975	0.006	0.824	115.600
**NF**	0.972	0.004	0.834	152.200

Table S3. EIS parameters of synthesized catalysts for HER.

Materials	Rs(Ω)	CPE-T	CPE-P	Rct(Ω)
**FeNi LDH/V_2CT_x/NF**	0.991	0.00077	0.850	10.85
**Pt/C/NF**	1.998	0.0074	0.833	8.963
**FeNi LDH/NF**	1.061	0.00028	0.902	12.36
**$Ni(OH)_2$/V_2CT_x/NF**	0.936	0.0006	0.875	24.73
**V_2CT_x/NF**	0.914	0.00115	0.868	67.4
**NF**	0.957	0.00109	0.826	106.1

Table S4. The current density of overall water splitting (50 mA cm^{-2}) values between FeNi LDH/V_2CT_x/NF in this work and various Ni/Fe-based catalysts recently reported.

Materials	Electrolyte	voltage（V）	Ref.
**FeNi LDH/V_2CT_x/NF**	1M KOH	1.74	This work

**$CoS_2/Ti_3C_2T_x$**	1M KOH	1.78	[19]
**$FeNi/Mo_2TiC_2T_x/NF$**	1M KOH	1.74	[20]
**NiFe LDH/NiCoP/NF**	1M KOH	1.75	[21]
**$NiCo_2S_4$@NiFe- LDH/NF**	1M KOH	1.83	[22]
**$CoNi_2S_4/Ni_3S_2$@NF**	1M KOH	1.83	[23]
**NiCo-LDH/NF**	1M KOH	1.86	[24]
**NiFe LDH/$Ni(OH)_2$/NF**	1M KOH	1.74	[25]

Table S5. Comparison of η_{10} and Tafel slope HER between FeNi LDH/V_2CT_x/NF in this work and various LDH catalysts recently reported.

Materials	η=10 mV	Tafel slope	Ref.
**FeNi LDH/V_2CT_x/NF**	151	136	This work
**NiFe LDH/NiCoP/NF**	145	88.2	[21]
**NiFeCo LDH/NF**	150	73	[26]
**A-NiCo LDH/NF**	151	57	[27]
**FeCo LDH/PANI/NF**	168	115	[28]
**MoP/NiCo-LDH/NF**	170	145	[29]
**$FeNi/Mo_2TiC_2T_x/NF$**	175	103.5	[20]

References

1. Yu, M.; Zhou, S.; Wang, Z.; Zhao, J.; Qiu, J. Boosting electrocatalytic oxygen evolution by synergistically coupling layered double hydroxide with MXene. *Nano Energy* **2018**, *44*, 181–190. https://doi.org/10.1016/j.nanoen.2017.12.003.
2. Ma, W.; Ma, R.; Wu, J.; Sun, P.; Liu, X.; Zhou, K.; Sasaki, T. Development of efficient electrocatalysts via molecular hybridization of NiMn layered double hydroxide nanosheets and graphene. *Nanoscale* **2016**, *8*, 1042510432. doi:10.1039/c6nr00988c.
3. Wang, W.; Liu, Y.; Li, J.; Luo, J.; Fu, L.; Chen, S. NiFe LDH nanodots anchored on 3D macro/mesoporous carbon as a high-performance ORR/OER bifunctional electrocatalyst. *J. Mater. Chem. A* **2018**, *6*, 14299–14306. doi:10.1039/c8ta05295f.
4. Jeong, S.; Hu, K.; Ohto, T.; Nagata, Y.; Masuda, H.; Fujita, J.-i.; Ito, Y. Effect of graphene encapsulation of NiMo alloys on oxygen evolution reaction. *ACS Catal.* **2019**, *10*, 792–799. doi:10.1021/acscatal.9b04134.
5. Wang, Y.; Xie, C.; Zhang, Z.; Liu, D.; Chen, R.; Wang, S. In Situ Exfoliated, N-Doped, and Edge-Rich Ultrathin Layered Double Hydroxides Nanosheets for Oxygen Evolution Reaction. *Adv. Funct. Mater.* **2018**, *28*, 1703363. doi:10.1002/adfm.201703363.
6. Teng, Y.; Wang, X.D.; Liao, J.F.; Li, W.G.; Chen, H.Y.; Dong, Y.J.; Kuang, D.B. Atomically thin defect-rich Fe-Mn-O hybrid nanosheets as high efficient electrocatalyst for water oxidation. *Adv. Funct. Mater.* **2018**, *28*, 1802463. doi:10.1002/adfm.201802463.
7. Fang, H.; Huang, T.; Liang, D.; Qiu, M.; Sun, Y.; Yao, S.; Yu, J.; Dinesh, M.M.; Guo, Z.; Xia, Y. Prussian blue analog-derived 2D ultrathin $CoFe_2O_4$ nanosheets as high-activity electrocatalysts

for the oxygen evolution reaction in alkaline and neutral media. *J. Mater. Chem. A* **2019**, *7*, 7328–7332. doi:10.1039/C9TA00640K.

8. Hu, L.; Li, M.; Wei, X.; Wang, H.; Wu, Y.; Wen, J.; Gu, W.; Zhu, C. Modulating interfacial electronic structure of CoNi LDH nanosheets with Ti_3C_2T MXene for enhancing water oxidation catalysis. *Chem. Eng. J.* **2020**, *398*, 125605. https://doi.org/10.1016/j.cej.2020.125605.
9. Du, C.F.; Sun, X.; Yu, H.; Fang, W.; Jing, Y.; Wang, Y.; Li, S.; Liu, X.; Yan, Q. $V_4C_3T_x$ MXene: A promising active substrate for reactive surface modification and the enhanced electrocatalytic oxygen evolution activity. *InfoMat* **2020**, *2*, 950–959. doi:10.1002/inf2.12078.
10. Wen, Y.; Wei, Z.; Liu, J.; Li, R.; Wang, P.; Zhou, B.; Zhang, X.; Li, J.; Li, Z. Synergistic cerium doping and MXene coupling in layered double hydroxides as efficient electrocatalysts for oxygen evolution. *J. Energy Chem.* **2021**, *52*, 412–420. doi:10.1016/j.jechem.2020.04.009.
11. Lu, Y.; Fan, D.; Chen, Z.; Xiao, W.; Cao, C.; Yang, X. Anchoring Co_3O_4 nanoparticles on MXene for efficient electrocatalytic oxygen evolution. *Sci Bull* **2020**, *65*, 460–466. doi:10.1016/j.scib.2019.12.020
12. Tian, M.; Jiang, Y.; Tong, H.; Xu, Y.; Xia, L. MXene-Supported FeCo-LDHs as Highly Efficient Catalysts for Enhanced Electrocatalytic Oxygen Evolution Reaction. *ChemNanoMat* **2019**, *6*, 154–159. https://doi.org/10.1002/cnma.201900613.
13. Liu, Q.; Zhou, F.; Bai, Y.; Hu, W. Evaluating Properties of Carbon-Free Nano-NiCoFe-LDHs with Molybdate as Oxygen Evolution Catalysts and Their Applications in Rechargeable Air Electrodes. *Energ Fuel* **2021**, *35*, 20374–20385. doi:10.1021/acs.energyfuels.1c03475.
14. Chen, J.; Long, Q.; Xiao, K.; Ouyang, T.; Li, N.; Ye, S.; Liu, Z.-Q. Vertically-interlaced NiFeP/MXene electrocatalyst with tunable electronic structure for high-efficiency oxygen evolution reaction. *Sci. Bull* **2021**, *66*, 1063–1072. doi:10.1016/j.scib.2021.02.033.
15. Chen, Y.; Yao, H.; Kong, F.; Tian, H.; Meng, G.; Wang, S.; Mao, X.; Cui, X.; Hou, X.; Shi, J. V_2C MXene synergistically coupling FeNi LDH nanosheets for boosting oxygen evolution reaction. *Appl. Catal. B Environ.* **2021**, *297*, 120474. https://doi.org/10.1016/j.apcatb.2021.120474
16. Chen, S.; Yu, C.; Cao, Z.; Huang, X.; Wang, S.; Zhong, H. Trimetallic NiFeCr-LDH/MoS_2 composites as novel electrocatalyst for OER. *Int. J. Hydrogen Energ.* **2021**, *46*, 7037–7046. doi:10.1016/j.ijhydene.2020.11.249.
17. Hao, C.; Wu, Y.; An, Y.; Cui, B.; Lin, J.; Li, X.; Wang, D.; Jiang, M.; Cheng, Z.; Hu, S. Interface-coupling of CoFe-LDH on MXene as high-performance oxygen evolution catalyst. *Mater. Today Energy* **2019**, *12*, 453–462. doi:10.1016/j.mtener.2019.04.009.
18. Ye, C.; Zhang, L.; Yue, L.; Deng, B.; Cao, Y.; Liu, Q.; Luo, Y.; Lu, S.; Zheng, B.; Sun, X. A NiCo LDH nanosheet array on graphite felt: an efficient 3D electrocatalyst for the oxygen evolution reaction in alkaline media. *Inorg. Chem. Front.* **2021**, *8*, 3162–3166. doi:10.1039/d1qi00428j.
19. Han, S.; Chen, Y.; Hao, Y.; Xie, Y.; Xie, D.; Chen, Y.; Xiong, Y.; He, Z.; Hu, F.; Li, L.; et al. Multi-dimensional hierarchical CoS_2@MXene as trifunctional electrocatalysts for zinc-air batteries and overall water splitting. *Sci. China Mater.* **2020**, *64*, 1127–1138. doi:10.1007/s40843-020-1524-5.
20. Wang, J.; He, P.; Shen, Y.; Dai, L.; Li, Z.; Wu, Y.; An, C. FeNi nanoparticles on $Mo_2TiC_2T_x$ MXene@nickel foam as robust electrocatalysts for overall water splitting. *Nano Res.* **2021**, *14*, 3474–3481. doi:10.1007/s12274-021-3660-0.
21. Zhang, H.; Li, X.; Hähnel, A.; Naumann, V.; Lin, C.; Azimi, S.; Schweizer, S.L.; Maijenburg, A.W.; Wehrspohn, R.B. Bifunctional Heterostructure Assembly of NiFe LDH Nanosheets on NiCoP Nanowires for Highly Efficient and Stable Overall Water Splitting. *Adv. Funct. Mater.* **2018**, *28*, 1706847. https://doi.org/10.1002/adfm.201706847.
22. Liu, J.; Wang, J.; Zhang, B.; Ruan, Y.; Lv, L.; Ji, X.; Xu, K.; Miao, L.; Jiang, J. Hierarchical $NiCo_2S_4$@NiFe LDH Heterostructures Supported on Nickel Foam for Enhanced Overall-Water-Splitting Activity. *ACS Appl. Mater. Interfaces* **2017**, *9*, 15364–15372. https://doi.org/10.1021/acsami.7b00019.
23. Dai, W.; Ren, K.; Zhu, Y.-a.; Pan, Y.; Yu, J.; Lu, T. Flower-like $CoNi_2S_4$/Ni_3S_2 nanosheet clusters on nickel foam as bifunctional electrocatalyst for overall water splitting. *J. Alloy Compd.* **2020**, *844*, 156252. doi:10.1016/j.jallcom.2020.156252.

24. Liu, W.; Bao, J.; Guan, M.; Zhao, Y.; Lian, J.; Qiu, J.; Xu, L.; Huang, Y.; Qian, J.; Li, H. Nickel–cobalt-layered double hydroxide nanosheet arrays on Ni foam as a bifunctional electrocatalyst for overall water splitting. *Dalton Trans.* **2017**, *46*, 8372–8376. https://doi.org/10.1039/c7dt00906b.
25. Gultom, N.S.; Abdullah, H.; Hsu, C.-N.; Kuo, D.-H. Activating nickel iron layer double hydroxide for alkaline hydrogen evolution reaction and overall water splitting by electrodepositing nickel hydroxide. *Chem. Eng. J.* **2021**, *419*, 129608. doi:10.1016/j.cej.2021.129608.
26. Babar, P.; Lokhande, A.; Karade, V.; Pawar, B.; Gang, M.G.; Pawar, S.; Kim, J.H. Bifunctional 2D Electrocatalysts of Transition Metal Hydroxide Nanosheet Arrays for Water Splitting and Urea Electrolysis. *ACS Sustain. Chem. Eng.* **2019**, *7*, 10035–10043. doi:10.1021/acssuschemeng.9b01260.
27. Yang, H.; Chen, Z.; Guo, P.; Fei, B.; Wu, R. B-doping-induced amorphization of LDH for large-current-density hydrogen evolution reaction. *Appl. Catal. B-Environ.* **2020**, *261*, 118240. doi:10.1016/j.apcatb.2019.118240.
28. Han, X.; Lin, Z.; He, X.; Cui, L.; Lu, D. The construction of defective FeCo-LDHs by in-situ polyaniline curved strategy as a desirable bifunctional electrocatalyst for OER and HER. *Int. J. Hydrogen Energy* **2020**, *45*, 26989–26999. https://doi.org/10.1016/j.ijhydene.2020.07.006.
29. Wang, T.; Wu, H.; Feng, C.; Zhang, L.; Zhang, J. MoP@NiCo-LDH on nickel foam as bifunctional electrocatalyst for high efficiency water and urea–water electrolysis. *J. Mater. Chem. A* **2020**, *8*, 18106–18116. https://doi.org/10.1039/d0ta06030e.
